# COVID Stress, socioeconomic deprivation, and intimate partner aggression during the COVID-19 pandemic

**DOI:** 10.1186/s12889-022-14093-w

**Published:** 2022-09-02

**Authors:** Julia F. Hammett, Miklós B. Halmos, Dominic J. Parrott, Cynthia A. Stappenbeck

**Affiliations:** 1 Edson College of Nursing and Health Innovation, Arizona State University, 500 N. 3rdSt, Phoenix, AZ 85004 USA; 2 Department of Psychology, Georgia State University, Atlanta, GA USA

**Keywords:** COVID-19, Intimate partner aggression, Socioeconomic deprivation, Stress

## Abstract

**Background:**

Intimate partner aggression (IPA) is a prevalent public health concern that is associated with multiple negative consequences. Rates of IPA in the U.S. have increased since the onset of the Coronavirus Disease 2019 (COVID-19) pandemic, likely due to stress associated with the pandemic. Socioeconomic deprivation is associated with COVID-19 outcomes as well as IPA. However, whether socioeconomic deprivation interacts with COVID-19 stress in predicting IPA remains unclear.

**Methods:**

Using a sample of 510 individuals recruited via Qualtrics Research Services in April 2020, the present study tested whether socioeconomic deprivation moderates the association between COVID-19 stress and IPA perpetration and victimization. Participants completed a questionnaire battery that included measures of COVID-19 stressors and physical and psychological IPA perpetration and victimization. In addition, participants reported their residential zip codes, which were subsequently matched with scores on the Social Deprivation Index, a composite measure of seven demographic variables from the 5-year American Community Survey.

**Results:**

Sequential generalized linear models in Mplus Version 8.7 showed that the effects of COVID-19 stress on physical IPA perpetration and psychological IPA victimization can be best understood through its interactive effects with socioeconomic deprivation. Higher COVID-19 stress was associated with higher levels of physical IPA perpetration and psychological IPA victimization when socioeconomic deprivation was low but not when socioeconomic deprivation was high. Importantly, however, overall rates of IPA were higher among individuals with higher socioeconomic deprivation than among individuals with lower socioeconomic deprivation, regardless of the amount of COVID-19 stress they experienced.

**Conclusions:**

The present analyses implicate COVID-19 stress as a critical correlate of IPA and show that the association between this stress and physical IPA perpetration and psychological IPA victimization may be particularly salient among individuals who live in areas of lower socioeconomic deprivation. Furthermore, our results clearly pinpoint the detrimental effects of socioeconomic deprivation more broadly, showing that individuals who live in more deprived areas tend to have high levels of IPA regardless of their level of COVID-19 stress. These findings call for public health policies at the community and societal level that target not only COVID-related stress but also the impacts of socioeconomic inequality.

## Background

Intimate partner aggression (IPA), including both physical and psychological aggression, is highly prevalent, with more than 10 million adults in the United States (U.S.) experiencing IPA every year [1]. The lasting consequences of IPA for individuals’ health and wellbeing are unambiguous and include increased risk for substance misuse and symptoms of depression and posttraumatic stress disorder [2, 3]. In U.S., IPA costs $8.3 billion annually, including medical costs ($4.1 billion) and lost productivity ($1.8 billion) [4–6]. Because long-term physical and mental health consequences are common following IPA [3, 7, 8], these high annual health care costs can persist for as long as 15 years after the abuse [9]. Moreover, data from the National Violence Against Women Survey suggest that survivors of IPA lose approximately 8 million days of paid work [5], placing these individuals at significant economic disadvantage, which can in turn further increase economic disparities between survivors and non-survivors.

Extant reports suggest that rates of IPA in the U.S. have increased since the onset of the pandemic associated with Coronavirus Disease 2019 (COVID-19) [10], likely due to the immense stressors (referred to hereafter as *COVID-19 stress*) directly resulting from the pandemic’s profound impacts [11]. Socioeconomic deprivation, such as living in areas with high rates of poverty, unemployment, and lack of education, is strongly associated with COVID-19 outcomes [12] as well as IPA [13, 14]. However, the effect of socioeconomic deprivation on the positive association between COVID-19 stress and IPA is unclear. Knowing how COVID-19 stress and socioeconomic deprivation interact to predict IPA is crucial from an intervention and policy standpoint aimed at determining which resources and services should be allocated to certain populations. Therefore, the present study examined direct and interactive associations between COVID-19 stress and socioeconomic deprivation on IPA perpetration and victimization among a sample of diverse U.S. adults who provided data at the height of mandatory COVID-19 shelter-in-place restrictions in April 2020.

### Application of the vulnerability-stress-adaption model to COVID-19 stress, socioeconomic deprivation, and IPA

A useful framework for organizing factors believed to affect risk for IPA is the Vulnerability-Stress-Adaption (VSA) model [15]. According to this model, a combination of *enduring vulnerabilities* and *external stressors* influence relationship outcomes by affecting individuals' abilities to collaboratively adapt to stressors and solve problems (i.e., *adaptive processes*). IPA can be thought of as a maladaptive process, and thus a more likely outcome when individuals are vulnerable and stressed.

Socioeconomic deprivation represents an enduring vulnerability that might be associated with greater risk for IPA by influencing the content of intimate partners' discussions, facilitating negative emotions, and fostering norms of aggression. Couples who live in socioeconomically deprived neighborhoods may be more prone to discussions of negatively charged topics such as paying bills or putting food on the table [16]. Moreover, these couples may believe that aggression is a more acceptable form of communication due to increased exposure to violence in their environments [17, 18]. Regarding stressors, COVID-19 and its associated social distancing measures have led to a variety of negative economic, social, and psychological impacts on people’s daily lives. The collective impact of these negative outcomes has caused significant stress in individuals from all socioeconomic backgrounds. According to the VSA model, COVID-19 stress is predicted to interact with enduring vulnerabilities (i.e., socioeconomic deprivation) to determine individuals’ risk for IPA. However, the direction of this interactive effect is unclear.

On the one hand, it is possible that the positive association between COVID-19 stress and IPA is exacerbated by higher levels of socioeconomic deprivation, such that this association is stronger under conditions of high, relative to low, deprivation. As such, individuals living in areas with higher socioeconomic deprivation may find themselves especially overwhelmed by the stressors associated with COVID-19 and as a result, may be more prone to resort to and experience aggression in their relationships. On the other hand, an alternative possibility holds that COVID-19 stress and IPA are positively associated at low, but not high, levels of socioeconomic deprivation. Here, it is posited that individuals living in areas with higher socioeconomic deprivation already experience higher stress overall; thus, the newly added stressors resulting from COVID-19 may not add appreciably to the already high risk of IPA that these individuals may experience. However, this association would be evident among individuals living with lower socioeconomic deprivation, as their reduced vulnerability and consequent lower levels of general external stress – would leave “more room” for COVID-19 stress to exert an impact. In line with this latter supposition, research has shown that individuals with higher education and the highest levels of income experienced greater decreases in life satisfaction from before to during the COVID-19 pandemic in comparison to those with lower education and income [19].

In summary, although the VSA model suggests that COVID-19 stress and socioeconomic deprivation should interact in predicting risk for IPA, the direction of this interaction is not clear, raising questions about policy efforts for resource allocation. If COVID-19 stress leads to particularly negative outcomes among individuals who live in areas of greater socioeconomic deprivation (Hypothesis 1), provision of resources and support during and after the pandemic should be concentrated among these populations. However, if COVID-19 stress leads to particularly negative outcomes among individuals who live in areas of less socioeconomic deprivation or if the effect of COVID-19 stress on IPA is independent of socioeconomic deprivation (Hypothesis 2), resources to combat the negative effects of the pandemic should be allocated towards all segments of society.

### The present study

The present study sought to resolve these competing hypotheses by testing whether the association between COVID-19 stress and IPA perpetration and victimization would vary depending on the level of socioeconomic deprivation in the areas where individuals were living. To achieve this goal, we surveyed 519 adults in the U.S. who had been in an intimate relationship for the past 6 months. Importantly, all participants completed the survey in April 2020, during the height of mandatory shelter-in-place restrictions across the U.S.

Because measures capturing area-level socioeconomic deprivation may be particularly useful tools in guiding policy efforts aimed at identifying areas for resource allocation, we used the Social Deprivation Index (SDI) [20] to measure socioeconomic deprivation. The SDI is a composite measure of area-level deprivation based on seven demographic characteristics (see Method section) collected in the American Community Survey. The SDI aims to quantify levels of socioeconomic deprivation across small geographic areas, evaluate their associations with health outcomes, and address health inequities. Using an established measure composed of multiple indices tapping into facets of socioeconomic deprivation also helps overcome biases associated with commonly used single-item self-report measures of socioeconomics (e.g., an individual's income), which have been shown to lead to inconsistent conclusions across studies [21].

## Method

The two competing hypotheses tested herein utilized data that were drawn from a larger investigation examining changes in interpersonal relations and substance use among individuals who were in romantic relationships during the initial months of the COVID-19 pandemic [11]. These hypotheses are novel and have not been tested previously, and the analytic plan was developed specifically to address these hypothesis. Although the present study did not examine effects of substance use, participants were required to meet alcohol consumption eligibility criteria for the parent study (see below). Due to the larger investigation’s hypotheses focused on exploring interactive effects of COVID-19 stress and minority stress on IPA (e.g., see [22]), individuals who self-identified as a sexual or gender minority were oversampled. Measures pertinent to the current study were administered as part of a larger questionnaire battery.

### Participants

Data were collected from 519 individuals. Eligibility criteria included being at least 18 years old, being in a current romantic relationship for at least 6 months, and consuming at least one alcoholic beverage in the last month. Eight participants were excluded from analyses because they did not reside in the United States at the time of participation, and one was excluded because they failed to pass response validity and quality checks, resulting in a final analytic sample of 510 participants. About half of the participants (50.8%) endorsed a sexual or gender minority identity, 59.4% reported being assigned female at birth, and 49.2% endorsed a heterosexual orientation. The majority of participants identified as either a woman (57.2%) or a man (40.4%), with few participants who endorsed a nonbinary (1.2%), trans (0.6%), or other (0.6%) gender identity. Participants ranged in age from 18 to 76 years (*M* = 38.84, *SD* = 14.75). The racial composition consisted of 72% White, 9.4% Hispanic or Latinx, 9.2% Black or African American, 3.3% Asian, 2.9% who endorsed multiple racial identities, and 3.2% who endorsed other racial identities. Participants reported having a mean yearly income of $43,313 (*SD* = $22,597) and having completed 15.76 years (SD = 3.80) of education. They had been in their current relationship for 11.47 years (*SD* = 12.02 years) on average, and reported seeing their current partner in person on 6.39 (*SD* = 1.36) days per week overall and on 6.15 (*SD* = 1.94) days per week during the shelter-in-place period. Please see Table 1 for additional information on sample characteristics.

**Table 1:** Sample characteristics

Variables	N	Min	Max	Mean	SD	%Yes
Age (Years)	510	18.00	76.00	38.84	14.75	–
Yearly Income ($)	498	2,500.00	70,000.00	43,313.25	22,597.12	–
Education (Years)	510	2.00	34.00	15.76	3.80	–
Relationship Length (Months)	510	0.50	50.67	11.47	12.02	–
Days/Week Seeing Partner (Overall)	508	0.00	7.00	6.38	1.37	–
Days/Week Seeing Partner (COVID)	510	0.00	7.00	6.14	1.95	–
COVID-19 Stress	510	2.00	29.00	11.00	4.83	–
Socioeconomic Deprivation	494	1.00	100.00	53.19	28.76	–
Physical IPA Perpetration	510	0.00	23.00	0.74	2.46	18.50
Physical IPA Victimization	510	0.00	29.00	0.88	2.92	18.80
Psychological IPA Perpetration	506	0.00	90.00	5.44	10.26	67.10
Psychological IPA Victimization	507	0.00	77.00	5.39	11.46	65.00

*Note. COVID-19* Coronavirus Disease 2019, *IPA* Intimate Partner Aggression, *Min* Observed Minimum, *Max* Observed Maximum, *SD* Standard Deviation

### Procedure

All data were collected from April 16, 2020 to April 23, 2020, which corresponded with the height of shelter-in-place restrictions of the COVID-19 pandemic that started in late-March 2020 in most U.S. states. Participants were recruited using Qualtrics Research Services, which provides an online research panel service. Qualtrics released the study to panel members and provided compensation consistent with their participation agreement. Quotas were set based on key demographics to ensure we obtained a representative sample based on age, sex assigned at birth, sexual and gender identity, household income, and race/ethnicity. The research panel from which the sample was drawn has been shown to be representative of the U.S. population with respect to demographics [23].

Interested individuals were provided an online consent form. Those who provided consent were asked to complete a single online survey hosted by Qualtrics, including screening questions to establish eligibility and determine quotas. Individuals who did not meet eligibility criteria or reported a demographic characteristic for which the quota was met were immediately exited from the survey. All procedures were approved by the University’s Institutional Review Board.

### Measures

#### COVID-19 stress

Modified versions of the Pandemic Stress Index [24] and COVID-19 Protective Actions and Interruptions to Care [25] were administered to assess COVID-19 stress. Participants indicated whether or not (0 = *no*, 1 = *yes*) the COVID-19 pandemic impacted their life or behavior (e.g., practicing social distancing, isolating or quarantining) and whether they had experienced various physical (e.g., more or less sleep), psychological (e.g., anxiety, depression), social (e.g., missed or postponed major life event/milestone), economic (e.g., unemployment, inadequate childcare), and health-related stressors (e.g., being diagnosed or hospitalized with COVID-19, fear of getting or giving COVID-19 to someone else) during COVID-19. A total score representing participants’ overall endorsement of COVID-19 stress was computed by summing the 30 items. This total score exhibited a normal distribution (*M* = 10.99, *SD* = 4.83, Skew = 0.22, Kurtosis =  − 0.39) and good internal consistency (Cronbach’s α = 0.80).^1^

#### Socioeconomic deprivation

The SDI [20] was used to measure socioeconomic deprivation. The SDI is a composite measure of seven demographic variables from the 5-year American Community Survey, administered by the U.S. Census Bureau. The variables included in the index are: 1) percent living in poverty; 2) percent with less than 12 years of education; 3) percent single-parent households; 4) percent living in a rented housing unit; 5) percent living in an overcrowded housing unit; 6) percent of households without a car; and 7) percent non-employed adults under 65 years of age. Residence areas are ranked from 1 (least deprived) to 100 (most deprived) in the U.S. SDI data are publicly available at the Robert Graham Center’s website (https://www.graham-center.org/maps-data-tools/social-deprivation-index.html), and SDI scores can be downloaded at the Primary Care Service Area, county, census tract, and Postal Zip Code Tabulation Area level. Participants in the present study reported the postal zip code of their current residence. These zip codes were then matched with Postal Zip Code Tabulation Area-level SDI scores. In the current sample, SDI scores ranged from 1 to 100 and were relatively normally distributed (*M* = 53.19, *SD* = 28.76, Skew =  − 0.06., Kurtosis =  − 1.19).

#### Intimate partner aggression

Select items from the Physical and Psychological Aggression subscales of the Revised Conflict Tactics Scale (CTS-2) [26] assessed IPA perpetration and victimization in the period of time between the implementation of shelter-in-place restrictions in participants’ local area and completing the survey. Physical IPA perpetration was assessed with two items (i.e., “I threw things, kicked, or hit something” and “I pushed, grabbed, or hit”) and psychological IPA perpetration was assessed with four items (i.e., “I yelled at my partner,” “I sulked or withdrew from my partner,” “I insulted or called my partner names,” “I made threats to my partner”). To assess physical and psychological victimization, participants reported whether they had experienced the same two physical and four psychological acts from their partner. Participants rated the frequency with which each act had occurred since the start of the COVID-19 pandemic in their area on 7-point scales (0 = This has not happened; 1 = 1 time; 2 = 2 times; 3 = 3–5 times; 4 = 6–10 times; 5 = 11–20 times; 6 = 20 or more times). Response options were recoded to the midpoint (e.g., 3–5 was recoded to 4) following CTS-2 scoring recommendations [26] and items within each subscale were summed. The CTS-2 has demonstrated strong validity in previous research [26].

### Data analytic strategy

To evaluate the effects of COVID-19 stress and socioeconomic deprivation on IPA, we estimated separate sequential generalized linear models for physical and psychological IPA perpetration and victimization in Mplus Version 8.7 [27] using a negative binomial distribution with a log link function, per best practice recommendations [28]. Missing data were handled using maximum likelihood (ML) estimation. For each of the four IPA outcomes (physical IPA perpetration and victimization and psychological IPA perpetration and victimization), we first ran a main effects model including COVID-19 stress and socioeconomic deprivation. Then, we added interaction terms obtained from cross products of COVID-19 stress by socioeconomic deprivation to sequential models. Participants’ age, endorsement of a sexual minority identity (0 = *no*, 1 = *yes*), and yearly income were entered as covariates in all models. Significant interactions were interpreted using simple slopes analyses with values of socioeconomic deprivation set at one standard deviation above and below the mean [29]. Results are reported as Incidence Rate Ratios (IRR; i.e., exponentiated coefficients) which may be interpreted as the rate change in the dependent variable for every unit increase in the independent variable.

### Power analyses

Post-hoc Monte Carlo simulation analyses were conducted to determine whether the current analyses were adequately powered to detect small-to-medium effects (Cohen’s *f* = 0.2) of COVID-19 stress, socioeconomic deprivation, and their interaction on IPA. Results of the simulations indicated that a sample size of 510 participants would yield sufficient power of 0.93 to 0.95.

## Results

Descriptive statistics and correlations of all study variables are presented in Tables 1 and 2. The three covariates were not significantly associated with any of the dependent variables in the study analyses, with the exception of two significant negative correlations between age and physical and psychological IPA perpetration.

**Table 2:** Correlations of all study variables (*N* = 510)

Study Variables	1	2	3	4	5	6	7	8	9
1. Physical IPA Perpetration	1								
2. Physical IPA Victimization	0.69***	1							
3. Psychological IPA Perpetration	0.67***	0.59***	1						
4. Psychological IPA Victimization	0.49***	0.76***	0.72***	1					
5. COVID-19 Stress	0.07	.11**	0.24***	0.21***	1				
6. Socioeconomic Deprivation	0.07	0.05	0.08	0.04	0.04	1			
7. Age	-0.12**	-0.05	-0.12**	-0.05	-0.25***	-0.17**	1		
8. Sexual Minority Status	-0.05	-0.03	-0.01	0.01	0.20***	-0.05	-0.22***	1	
9. Yearly Income	-0.01	-0.03	-0.01	-0.06	-0.05	-.16**	.16***	0.03	1
***p < .001, **p < .01, *p < .05									

*Note. COVID-19* Coronavirus Disease 2019, *IPA* Intimate Partner Aggression. Sexual Minority Status was binary coded (0 = no, 1 = yes)

### Effects of COVID-19 Stress and Socioeconomic deprivation on intimate partner aggression

#### Physical IPA

**Fig. 1:**
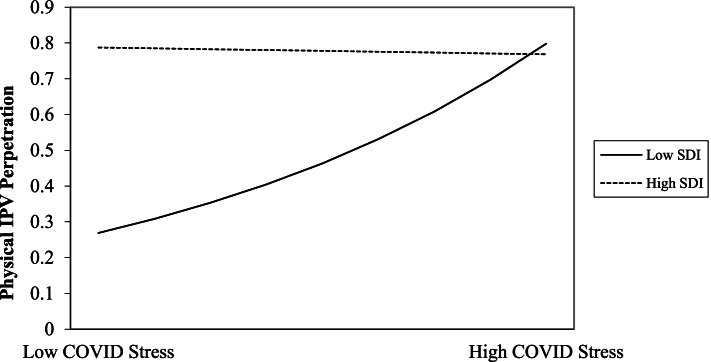
Interaction of COVID-19 stress by socioeconomic deprivation predicting physical IPA perpetration. *Note.* High/low plot points indicate 1 SD above and below the mean, respectively. COVID-19 = Coronavirus Disease 2019; IPA = Intimate Partner Aggression; SDI = Social Deprivation Index

As can be seen in Table 3, neither the COVID-19 stress nor the socioeconomic deprivation main effect on physical IPA perpetration were statistically significant. However, there was a significant COVID-19 stress X socioeconomic deprivation interaction associated with physical IPA perpetration. Simple slope analyses (see Table 3) revealed that COVID-19 stress was significantly and positively associated with physical IPA perpetration among individuals living in areas of lower socioeconomic deprivation; in contrast, this association was not significant among individuals living in areas of higher socioeconomic deprivation. Visual inspection of Fig. 1 shows that individuals living in areas of higher socioeconomic deprivation perpetrated physical IPA at high, yet stable, rates regardless of their level of COVID-19 stress. Among individuals living in areas of lower socioeconomic deprivation, rates of physical IPA perpetration increased from low to high levels of COVID-19 stress.

**Table 3:** Independent and interactive effects of COVID-19 stress and socioeconomic deprivation on intimate partner aggression

Variables	IPA Perpetration				IPA Victimization			
	IRR	B	p	95% CI	IRR	B	p	95% CI
**Physical IPA Main Effects Model**								
COVID-19 Stress	1.061	0.059	0.096	[0.059, 0.118]	1.063	0.061	0.051	[0.010, 0.112]
Socioeconomic Deprivation	1.007	0.007	0.179	[0.007, 0.016]	1.006	0.006	0.252	[-0.003, 0.016]
**Physical IPA Interaction Model**								
COVID-19 Stress	1.057	0.055	0.102	[0.000, 0.110]	1.060	0.058	0.050	[0.009, 0.107]
Socioeconomic Deprivation	1.009	0.009	0.104	[0.000, 0.018]	1.008	0.008	0.169	[-0.002, 0.017]
COVID-19 Stress X Socioeconomic Deprivation	0.998	-0.002	0.032	[-0.004, -0.001]	0.999	-0.001	0.155	[-0.003, 0.000]
**Psychological IPA Main Effects Model**								
COVID-19 Stress	1.102	0.097	0.000	[0.071, 0.123]	1.104	0.099	0.000	[0.070, 0.127]
Socioeconomic Deprivation	1.004	0.004	0.150	[-0.001, 0.008]	1.006	0.006	0.036	[0.001, 0.011]
**Psychological IPA Interaction Model**								
COVID-19 Stress	1.102	0.097	0.000	[0.071, 0.122]	1.115	0.109	0.000	[0.081, 0.137]
Socioeconomic Deprivation	1.004	0.004	0.128	[0.000, 0.008]	1.008	0.008	0.007	[0.003, 0.012]
COVID-19 Stress X Socioeconomic Deprivation	0.999	-0.001	0.191	[-0.001, 0.000]	0.998	-0.002	0.000	[-0.003, -0.001]

*Note. COVID-19* Coronavirus Disease 2019, *IPA* Intimate Partner Aggression, *IRR* Incidence Rate Ratio, representing the rate of change in IPA associated with each one unit increase in the predictor variable. Models were estimated using a negative binomial distribution with a log link function. Unstandardized regression coefficients are shown

Analyses did not detect significant main effects of COVID-19 stress or socioeconomic deprivation. There also was no significant COVID-19 stress X socioeconomic deprivation interaction on physical IPA victimization (see Table 3).

#### Psychological IPA

As can be seen in Table 3, there was a statistically significant main effect of COVID-19 stress on psychological IPA perpetration, suggesting that individuals who experienced greater COVID-19 stress perpetrated psychological IPA at higher rates, regardless of the level of socioeconomic deprivation to which they were exposed. The main effect of socioeconomic deprivation and the COVID-19 stress X socioeconomic deprivation interaction effect on psychological IPA perpetration were not statistically significant.

**Fig. 2:**
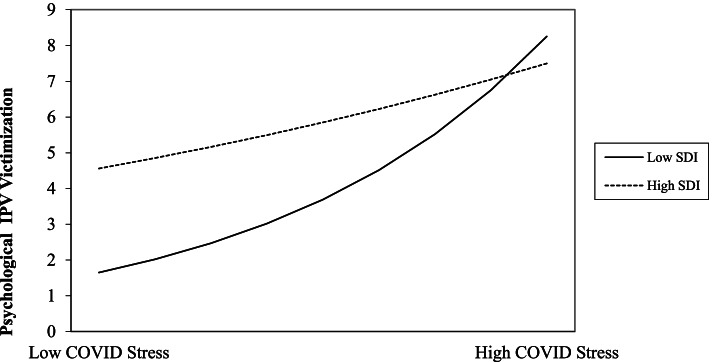
Interaction of COVID-19 Stress by Socioeconomic Deprivation Predicting Psychological IPA Victimization. *Note.* High/low plot points indicate 1 SD above and below the mean, respectively. COVID-19 = Coronavirus Disease 2019; IPA = Intimate Partner Aggression; SDI = Social Deprivation Index

Finally, both the COVID-19 stress and socioeconomic deprivation main effects on psychological IPA victimization were statistically significant, showing that individuals who experienced higher COVID-19 stress (regardless of socioeconomic deprivation) and who lived in more socioeconomically deprived areas (regardless of COVID-19 stress) experienced psychological IPA victimization at higher rates. Additionally, there was a significant COVID-19 stress X socioeconomic deprivation interaction on psychological IPA victimization (see Table 3). Simple slope analyses (see Table 4) revealed that COVID-19 stress was significantly and positively associated with psychological IPA victimization among individuals living in areas of both lower and higher socioeconomic deprivation. Although the association between COVID-19 stress and psychological IPA victimization was positive for individuals in both low and high areas of socioeconomic deprivation, this positive association was significantly stronger for individuals living in areas of lower (rather than higher) socioeconomic deprivation (Fig. 2).

**Table 4:** Simple Slope Effects of Significant Interactions

Interaction	IRR	B	p	95% CI
**COVID-19 Stress on Physical IPA Perpetration**				
At Low Socioeconomic Deprivation	1.125	0.118	0.008	[0.045, 0.191]
At High Socioeconomic Deprivation	0.991	-0.009	0.843	[-0.083, 0.065]
**COVID-19 Stress on Psychological IPA Victimization**				
At Low Socioeconomic Deprivation	1.182	0.167	0.000	[0.125, 0.209]
At High Socioeconomic Deprivation	1.052	0.051	0.014	[0.017, 0.085]

*Note. COVID-19* Coronavirus Disease 2019, *IPA* Intimate Partner Aggression, *IRR* Incidence Rate Ratio, representing the rate of change in IPA associated with each one unit increase in the predictor variable. Models were estimated using a negative binomial distribution with a log link function. Unstandardized regression coefficients are shown

## Discussion

This study examined direct and interactive associations between COVID-19 stress and area-level socioeconomic deprivation on IPA perpetration and victimization. Using a multimethod approach that combined self-report data on stress and aggression with more objective assessments of residential socioeconomic deprivation, the present research resulted in several key findings. First, COVID-19 stress was significantly and positively associated with psychological IPA perpetration and victimization, even after controlling for the effects of socioeconomic deprivation. Main effects of COVID-19 stress on physical IPA perpetration and victimization, over and above effects of socioeconomic deprivation, were not statistically significant. Second, area-level socioeconomic deprivation was positively associated with psychological IPA victimization after controlling for the effects of COVID-19 stress. Third, the association between COVID-19 stress and physical IPA perpetration and psychological IPA victimization can be best understood in the context of socioeconomic deprivation. High levels of socioeconomic deprivation were associated with high and relatively stable levels of physical IPA perpetration and psychological IPA victimization across all levels of COVID-19 stress. In contrast, at low levels of socioeconomic deprivation, the association between COVID-19 stress and physical IPA perpetration and psychological IPA victimization was significantly more positive. These results reify that overall, individuals who are the most socioeconomically vulnerable are disproportionately affected by IPA [30]. Only when faced with unusual circumstances – such as the sudden stress surrounding the COVID-19 pandemic – did less socioeconomically deprived individuals exhibit levels of IPA perpetration and victimization that resemble their more deprived counterparts.

The current findings provide partial support for the interactive vulnerability-by-stress effects predicted by the VSA model [15]. However, rather than demonstrating an exacerbation effect of socioeconomic deprivation, our results show that individuals residing in areas of higher socioeconomic deprivation were *less* affected by COVID-19-specific stress than those residing in areas of lower socioeconomic deprivation. Perhaps this is because individuals residing in areas of higher socioeconomic deprivation were already experiencing high rates of physical IPA perpetration and psychological IPA victimization, and this remained true regardless of their COVID-19 stress. Additionally, these effects are in line with at least some prior research showing that (a) individuals with higher education experienced a greater increase in depressive symptoms and a greater decrease in life satisfaction during COVID-19 in comparison to those with lower education, and (b) individuals at the highest levels of income experienced a greater decrease in life satisfaction during COVID-19 than individuals with lower levels of income [19].

It is possible that individuals from more socioeconomically deprived areas did not perceive the stressors associated with COVID-19 as severely because they were accustomed to enduring high levels of overall stress (e.g., related to living in areas with fewer resources and greater financial insecurity). In contrast, the sudden stressful life changes of the pandemic may have had a stronger impact on individuals from areas with less socioeconomic deprivation, who were less familiar with such hardship, compromising their conflict management skills and increasing their risk for IPA. Moreover, individuals from areas of higher socioeconomic deprivation may have been more likely to be front line workers [31] whose hourly wage, at least in some instances, may have even increased early in the pandemic (e.g., hazard pay), whereas individuals from areas of lower socioeconomic deprivation may have been more likely to be working at home [32]. Although front line work tends to be associated with higher potential exposure to the virus and thus higher potential COVID-19 stress, spending time at work also meant less time at home for these individuals, and hence less time in close proximity with their intimate partner with fewer opportunities for disagreements. Individuals residing in less socioeconomically deprived areas, on the other hand, may have had to spend more time in close contact with their partners, which, exacerbated by the uncertainty and stress of the pandemic, may have provided them with more chances to lash out in the heat of an argument and resort to or experience IPA in their relationship. Future research should examine whether these proposed explanations are accurate.

### Limitations and future directions

This study evidences several strengths, including the use of a large and diverse sample, assessment of COVID-19 stress and its correlates during the height of the shelter-in-place requirements early in the pandemic when concerns about the potential impact on IPA were high, and the use of a multimethod approach. However, current results should be interpreted in light of some limitations which also highlight important directions for future research. First, this study used a cross-sectional design and although we can be fairly certain that COVID-19 stress was proximally associated with IPA due to the restricted timeframe for reporting, it is not known whether this stress was causally related to IPA. Future research on the effects of stress associated with pandemics and/or natural disasters should consider daily diary or ecological momentary assessment to better inform causal connections. Relatedly, the current cross-sectional assessment only captured the early months of the pandemic (i.e., behaviors that had occurred between the onset of the pandemic in March 2020 and data collection in April 2020); thus, we do not know how COVID-19 stress and socioeconomic deprivation would continue to impact IPA throughout the course of the pandemic. It is likely that continued stressors of the ongoing pandemic may have impacted individuals differently. For example, factors such as the removal of hazard pay for essential workers, the closure of businesses that relied on in-person patronage, and the increase in prices for common goods and services may have placed a disproportionately greater strain on individuals in areas of low SDI who did not have the financial resources to absorb the effects of job losses, increased costs, etc.

Next, our measure of socioeconomic deprivation, the SDI, was matched with participant data on the basis of self-reported postal zip codes. In addition to potential misreports of zip code data, it is important to note that postal zip codes may span across relatively large demographic areas, and neighborhoods within each zip code can be quite heterogenous with regards to a variety of socioeconomic indicators. Additionally, the SDI focuses specifically on socioeconomic deprivation whereas additional indices exist that more broadly assess social vulnerability (e.g., CDC/ATSDR Social Vulnerability Index, Census Bureau Community Resilience Estimates) include measures of minority status, language barriers, and broadband internet access in addition to the socioeconomic indicators captured by the SDI. The broader indices of social vulnerability that we are aware of are connected to one’s census tract and would require more specific address information than we collected to adequately link participants’ neighborhood to those indices. We recommend that future research collect address data necessary to identify census tract to assess the interactive impact of COVID-19 stress and a broad index of social vulnerability on IPA.

This research used an abbreviated version of the CTS-2 to assess IPA. Although the smaller number of items enabled us to obtain a relatively large sample in a short period of time (thus tapping specifically into experiences during a critical phase of the COVID-19 pandemic), it may have prevented us from detecting the full range of potential IPA behaviors that occurred during this period. We hope that data collected from other researchers will provide a more complete assessment of the full range of IPA behaviors experienced during this critical time. Relatedly, collecting data on IPA and its correlates from only one member of the dyad prevented us from comparing reports of IPA rates across partners and from assessing not only actor but also partner effects of COVID-19 stress on aggression (see Actor Partner Interdependence Model [APIM]) [33]. Future research should take advantage of dyadic assessments to capture reports of perpetration and victimization from both partners to more fully elucidate patterns of IPA within intimate relationships. Finally, despite evidence of Qualtrics samples’ representativeness of national U.S. demographics, the current findings may not generalize to other types of samples, such as less diverse groups, teenagers, or older adults.

## Conclusions and implications

The present results implicate COVID-19 stress as a critical correlate of IPA and show that the association between this stress and physical IPA perpetration as well as psychological IPA victimization may be particularly salient among individuals who live in areas of lower socioeconomic deprivation. Furthermore, our findings clearly pinpoint the detrimental effects of socioeconomic deprivation more broadly, as individuals who lived in more deprived areas had high levels of IPA regardless of their level of COVID-19 stress. Because the economic, social, and psychological stress associated with COVID-19 are likely to endure even after the pandemic phase has ended, studies are needed that examine the proximal and causal association between COVID-19 stress, socioeconomic deprivation, and IPA. Replication of the present findings, potentially using experimental or longitudinal designs, would provide strong justification that interventions which target stress could be useful in reducing IPA.

Although not intended to directly inform IPA policy or intervention efforts during a pandemic, the present findings suggest that focusing on couples’ stress will be critical. Our results show that individuals living in areas of lower socioeconomic deprivation are particularly vulnerable to the detrimental effects of COVID-19 stress on relationship functioning in that their levels of IPA resemble those of individuals living in areas of greater deprivation when stress is high. These findings call for public health policies at the community and societal level that target not only COVID-related stress but also the impacts of socioeconomic inequality more broadly. Given IPA’s vast economic impact [4], socioeconomic determinants, such as the neighborhood-level deprivation effects examined in the present research, create a vicious cycle whereby those who live in socioeconomically deprived areas are at increased risk of experiencing IPA, and then will bear the economic impact of IPA (e.g., lost wages, increased health care costs) as a result. Structural economic interventions aimed at increasing supports to all individuals within a given community could have a significant impact in preventing IPA and thus stopping this cycle of aggression, violence, and deprivation [34].

## Data Availability

The datasets analyzed during the current study are available from the corresponding author on reasonable request.

## Abbreviations

APIM: Actor Partner Interdependence Model
COVID-19: Coronavirus Disease 2019
CTS2: Revised Conflict Tactics Scale
IPA: Intimate Partner Aggression
IRR: Incidence Rate Ratios
ML: Maximum Likelihood
SDI: Social Deprivation Index
U.S: United States
VSA: Vulnerability-Stress-Adaption

## Footnotes

1 Of note, the current measures of COVID-19 stress were deployed at the beginning of the pandemic. Because there are clear differences in timeline and associated impacts related to different points of the pandemic, results should be interpreted within the context of the start of the pandemic.”.
